# Pamphlets Received

**Published:** 1878-06

**Authors:** 


Suggestions in the Treatment of Spinal Diseases and Curvature.—By E. H. Coover, M.D., of Harrisburg, Pa.

---

Old Age.—Its Diseases and its Hygiene. By Lunsford P. Yandell, M.D., Louisville, Ky. (Reprint from *American Practitioner*, February, 1878.)

---

Eulogy—Upon Lunsford P. Yandell, M.D., Louisville, Ky. (Reprint from *American Practitioner*, April, 1878.)

---

The Iowa Catlin—for May, 1878. A Journal of Medicine and Surgery. Edited by Edward Lawrence, and published monthly at Osceola, Iowa. Terms $3 a year.

